# The normalized segment classification model: A new tool to compare spectral reflectance curves

**DOI:** 10.1002/ece3.6977

**Published:** 2020-11-13

**Authors:** Miguel Angel Rodríguez‐Gironés, Francismeire Jane Telles

**Affiliations:** ^1^ Estación Experimental de Zonas Áridas CSIC Almería Spain; ^2^ Programa de Pós‐Graduação em Ecologia e Conservação de Recursos Naturais Universidade Federal de Uberlândia Uberlândia Brazil

**Keywords:** brightness, color vision, perception, visual ecology, visual signals

## Abstract

1. Color patterns are complex traits under selective pressures from conspecifics, mutualists, and antagonists. To evaluate the salience of a pattern or the similarity between colors, several visual models are available. Color discrimination models estimate the perceptual difference between any two colors. Their application to a diversity of taxonomic groups has become common in the literature to answer behavioral, ecological, and evolutionary questions. To use these models, we need information about the visual system of our beholder species. However, many color patterns are simultaneously subject to selective pressures from different species, often from different taxonomic groups, with different visual systems. Furthermore, we lack information about the visual system of many species, leading ecologists to use surrogate values or theoretical estimates for model parameters.
2. Here, we present a modification of the segment classification method proposed by Endler (*Biological Journal of the Linnean Society*, 1990 *41*, 315–352): the normalized segment classification model (NSC). We explain its logic and use, exploring how NSC differs from other visual models. We also compare its predictions with available experimental data.
3. Even though the NSC model includes no information about the visual system of the receiver species, it performed better than traditional color discrimination models when predicting the output of some behavioral tasks. Although vision scientists define color as independent of stimulus brightness, a likely explanation for the goodness of fit of the NSC model is that its distance measure depends on brightness differences, and achromatic information can influence the decision‐making process of animals when chromatic information is missing.
4. Species‐specific models may be insufficient for the study of color patterns in a community context. The NSC model offers a species‐independent solution for color analyses, allowing us to calculate color differences when we ignore the intended viewer of a signal or when different species impose selective pressures on the signal.

## Graphical abstract

Color patterns are complex traits under a variety of biotic and abiotic selective pressures. In a community context, species‐specific models may be insufficient for the study of colour patterns. The NSC model offers a species‐independent solution for colour analyses, allowing ecologists to calculate colour differences when we ignore the intended viewer of a signal, or when different species impose selective pressures on the signal.

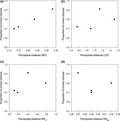

## 1 INTRODUCTION

Color patterns can decrease the predation probability of organisms by making them cryptic (Rodríguez‐Gironés & Maldonado, 2020) or displaying their unpalatability (Barnett et al., 2018; Su et al., 2015), they can advertise the quality of potential mates (Keyser & Hill, 2000; Siefferman & Hill, 2003) or lure prey (Vieira et al., 2017). To study the effect of colors on the behavior of animals, and the evolution of color patterns, we often need to quantify the extent to which one color differs from another.

After decades of intensive research, several color vision models have been proposed (reviewed in Renoult et al., 2017). When it is clear which species imposes selective pressures on a color pattern, we can use species‐specific color discrimination models to study the ecological role of the color pattern or its evolutionary trajectory. The color‐opponent coding model can be used for honeybees, *Apis mellifera* (Backhaus, 1991), the color hexagon model is a generalization for trichromatic Hymenoptera species (Chittka, 1992), and the receptor noise‐limited model can be used for any species (Vorobyev & Osorio, 1998). But what should we do if we ignore for which species a color signal is intended, or if several species impose selective pressures on a color pattern?

The strength of species‐specific color discrimination models is that they take into account the visual system of the viewer. All these models use species‐specific photoreceptor sensitivities to estimate the number of photons captured by different photoreceptor types, and the strength of the signal sent to higher information processing centers (Backhaus, 1991; Chittka, 1992; Vorobyev et al., 2001; Vorobyev & Osorio, 1998). In addition, the receptor noise‐limited model uses the noise level of the different photoreceptor channels (Vorobyev & Osorio, 1998). However, when different species, possibly having different color discrimination abilities, are interested in the signal – as can be the case of a caterpillar, coveted by wasps, spiders and birds, or when visual signals should be visible to conspecifics but cryptic to predators and prey – there is no species‐specific model we can apply. In these cases, rather than considering how viewers perceive the relevant colors, ecologists could compare the physical properties of light beams.

There are several equivalent ways of describing a light beam. Physicists will often describe light beams in terms of energy fluxes and frequencies, *ν* – specifying the amount of energy per unit time (power) in the frequency range (*ν*, *ν* + d*ν)* flowing through a unit of surface. Visual ecologists normally focus on photon fluxes and wavelengths, *λ*. Let us denote by *L*(*λ*)·d*λ* the spectral composition of a light beam – the number of photons flowing per unit time and unit surface with wavelength in the range (*λ*, *λ *+ d*λ*). Color is the perception of the spectral composition of light arriving to the retina – in a sense, the individual's perception of the shape of the function *L*(*λ*) (Kelber & Osorio, 2010; Kemp et al., 2015).

**Figure 1:**
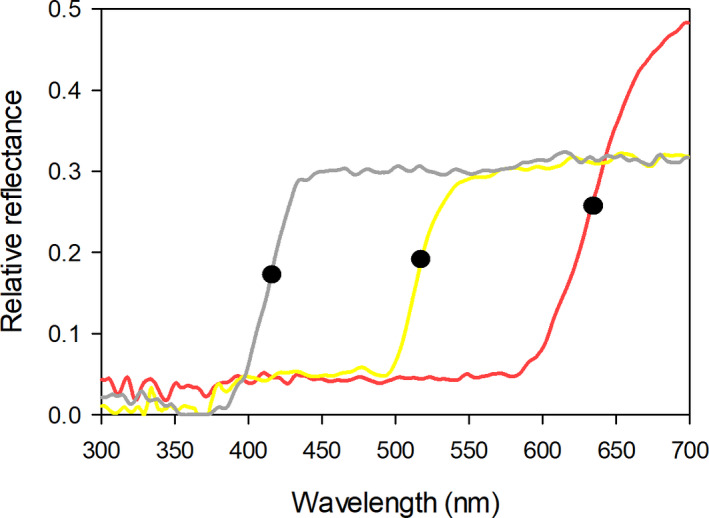
Normalized reflectance spectra of a human white flower (UV‐absorbing), *Begonia acida*; human yellow flower, *Cambessedesia ilicifolia*; and a human red flower (UV‐absorbing), *Ranunculus asiaticus*. Black dots indicate marker points (inflection points) calculated with the Spectral‐MP software (Dorin et al., 2020). Reflectance samples were obtained from FRED (Arnold et al., 2010)

Some biologically relevant colors can be characterized by the position of an inflection point in *L*(*λ*). Ultraviolet‐absorbing white, human yellow and red lights all have a sigmoidal *L*(*λ*) function and basically differ in the position of their inflection point: the wavelength at which *L*(*λ*) increases fastest (Figure 1). Because these functions have similar shapes and one can (more or less) be converted into another through a horizontal displacement, a single parameter, the wavelength corresponding to the inflection point, is sufficient to describe them. This is the reason why we can use the difference between the inflection points of two spectral composition curves as a measure of their overall similarity (Chittka & Menzel, 1992; Shrestha et al., 2014). Unfortunately, not all relevant colors can be characterized by a single inflection point. What humans perceive as greens have bell‐shaped spectral composition curves, and other colors have rather complex spectral composition curves, with several maxima and minima. As a result, the difference between the (main) inflection points of two spectral composition curves does not always provide a good indication of their shape difference.

Endler's (1990) segment classification scheme was one of the earliest attempts to measure differences in the shape of spectral composition curves, with the aim of providing an index of color similarity independent of the viewer's identity. In the next section, we review the segment classification scheme. We then present a modification of the model and compare its predictions with published experimental data, and with the predictions of alternative models.

## 2 THE SEGMENT CLASSIFICATION SCHEME

The segment classification scheme is a species‐independent method to transform the spectral distribution curve of a light beam into a set of three coordinates. The first two coordinates represent the chroma and hue of the light beam and the third coordinate, its brightness (Endler, 1990).

Let (*λ*_1_, *λ*_2_) be the range of wavelengths that the observer can perceive. A measure of brightness, *β*(*λ_a_*, *λ_b_*), over the wavelength segment (*λ_a_*, *λ_b_*) – that is, a measure of the total amount of light available in this range – is the flux of photons within this range reaching the eye from the object, which is proportional to the integral

(1) $$\beta \left(\lambda_{a},\lambda_{b}\right)=\int_{\lambda_{a}}^{\lambda_{b}}L\left(\lambda \right)\cdot d\lambda$$

The spectral distribution of the light impinging the eye when we view an object, *L*(*λ*), depends on the incident light, the physical properties of the object's surface, and the transmission properties of the medium. Pigments – molecules that preferentially absorb light with certain wavelengths – are responsible for the color of most objects. The surfaces of these objects are well characterized by their reflectance spectrum, *R*(*λ*), which gives the proportion of photons that they reflect as a function of their wavelength *λ*. For these objects, and ignoring the transmission properties of the medium,

(2) L(l)=R(l)·D(l),

where *D*(*λ*) is the spectral distribution of incident light.

When light scatter between the surface and the eye is important, as is the case in aquatic environments, in foggy conditions or even in clear atmosphere if the distances are very large, Equation (2) must incorporate the effect of scatter (Johnsen, 2012). The so‐called structural colors appear when the 3D structure of the surface leads to light interference (Parker, 2000). The physics of light interference and reflection are very different. In particular, for some structural colors, the wavelength of light impinging the eye depends on the angle at which the light beams leave the surface. For this reason, when we work with structural colors we cannot compute *L*(*λ*) from Equation (2). Instead, we must measure *L*(*λ*) directly or estimate it through some other means before using Equation (1) to compute *β*(*λ_a_*, *λ_b_*).

Similarly, the relative brightness over the interval can be defined as the brightness of the segment divided by the overall brightness, *β*(*λ_a_*, *λ_b_*)/*β*(*λ*_1_, *λ*_2_). With this definition of relative brightness, divide the entire range of visible light, (*λ*_1_, *λ*_2_), in four equally sized segments and call *B*, *G*, *Y*, and *R* the relative brightness of the first, second, third and fourth segments. The segment classification model is defined by three coordinate axes. Specifically, the coordinates (*X*_1_, *X*_2_, *X*_3_) of a color, corresponding to its chroma, hue and brightness, respectively, are.

(3a) $$X_{1}=\sqrt{{\left(R‐G\right)}^{2}+{\left(Y‐B\right)}^{2}}$$

(3b) $$X_{2}=\left\{\begin{array}{cc} \arcsin\left[\left(Y‐B\right)/X_{1}\right] & R\geq G,Y\geq B\\ \arcsin\left[\left(Y‐B\right)/X_{1}\right]+2\pi & R\geq G,Y<B\\ \pi ‐\arcsin\left[\left(Y‐B\right)/X_{1}\right] & R<G \end{array}\right)$$

(3c) $$X_{3}=b\left(l_{1},l_{2}\right).$$

From these coordinates, we can calculate perceptual distances between two colors. The chromatic distance is the Euclidean distance between the vectors (*X*_1_, *X*_2_), and the achromatic, brightness distance is the difference in *X*_3_ (Endler, 1990). The equation for *X*_2_ above differs from the original formulation, in which the arcsine function referred to its PASCAL implementation (Endler, personal communication). To generalize the expression, we have modified it as suggested by Smith (2014). It is important to stress that, because brightness is independent of photoreceptor spectral sensitivities, the segment classification scheme is species independent.

## 3 THE NORMALIZED SEGMENT CLASSIFICATION MODEL

### 3.1 Model development

We now use the segment classification scheme to construct a color discrimination model that is sensitive to brightness differences and is species independent. For vision scientists, this is an oxymoron: color differences exclude, by definition, brightness differences (Kelber et al., 2003). But in everyday English, color is “the property possessed by an object of producing different sensations on the eye as a result of the way it reflects or emits light”, (OUP, 2020) – a definition that certainly includes the brightness components and to which, as ecologists, we adhere.

Using as perceptual distance between two colors the Euclidean difference between the (*X*_1_, *X*_2_, *X*_3_) vectors corresponding to the two colors, with (*X*_1_, *X*_2_, *X*_3_) as originally defined (Endler, 1990), is unlikely to be of much use as a color discrimination model. The reason is that, with this definition, color distances would be dominated by the brightness component: in Equations ((3a), (3b), (3c)), (*X*_1_, *X*_2_) denotes a scale independent vector with length of order 1 (*B*, *G*, *Y*, and *R* are dimensionless positive numbers smaller than 1), while *X*_3_ is typically much larger. For instance, if we calculate *X*_3_ using the standard D65 illuminant (Wyszecki & Stiles, 1982) for *D*(*λ*) and the reflectance spectra of green leaves for *R*(*λ*), as given in the PAVO package (Maia et al., 2019), we obtain *X*_3_ = 424.7 if we group the spectral values in bins of 5 nm, and *X*_3_ = 2,102.7 if we use 1 nm bins. As a result, the Euclidean distance between the loci (*X*_1_, *X*_2_, *X*_3_) corresponding to two colors would typically be determined by their brightness difference (distance(*A*, *B*) ≈ Abs(*X*_3A_ − *X*_3B_)).

A useful distance measure requires that brightness differences are of the same order of magnitude as differences in hue and chroma. There are different ways to achieve this goal, and the Normalized Segment Classification color discrimination model, NSC, represents one of them. Because *X*_1_ and *X*_2_ are of order 1, to make sure that brightness differences do not swamp color differences we normalize the brightness component (*X*_3_ in Equation 3c). Let *β*_m_(*λ*_1_, *λ*_2_) be the maximum value that *β*(*λ*_1_, *λ*_2_) can achieve – that is, the value obtained for a white standard, with *R*(*λ*) = 1 in Equation (2) and hence *L*(*λ*) = *D*(*λ*) for all *λ* in (*λ*_1_, *λ*_2_). With this definition of *β*_m_, the coordinates in the NSC model, (*Z*_1_, *Z*_2_, *Z*_3_), of a stimulus are:

(4a) $$Z_{1}=R‐G$$

(4b) $$Z_{2}=Y‐B$$

(4c) $$Z_{3}=b\left(l_{1},l_{2}\right)\;/b_{m}\left(l_{1},l_{2}\right).$$

There are two important differences between coordinates (*X*_1_, *X*_2_, *X*_3_) and (*Z*_1_, *Z*_2_, *Z*_3_). First of all, (*X*_1_, *X*_2_) and (*Z*_1_, *Z*_2_) represent the same two‐dimensional vector, using different reference systems. Endler chose to represent the vector with polar coordinates (Smith, 2014) because in this way *X*_1_ represents the chroma and *X*_2_ the hue of a color. While this is an interesting property, it complicates the process of calculating distances: the Euclidean distance between the loci of colors *A* and *B*, represented in polar coordinates, is NOT given by the well‐known Pythagorean rule – it must be calculated using trigonometry instead. If we are interested in color differences, the Cartesian reference system (*Z*_1_ and *Z*_2_, Equation (4a), (4b), (4c)) is the natural choice. The second difference lies in the brightness coordinate: unlike *X*_3_, which can take very large values, *Z*_3_ in the NSC model ranges between 0 (black) and 1 (white).

The next step in the development of the NSC model is to generalize Equation (4a), (4b), (4c). As we will see, Equations ((3a), (3b), (3c)) and ((4a), (4b), (4c)) are inspired in the color perception of a trichromatic species. Before we can generalize them to di‐ or tetrachromatic species, we need to understand the relationship between color perception and the segment classification scheme.

The vast majority of species studied so far rely on color opponency for color perception. Color‐opponent mechanisms compare the output from several photoreceptor types. Let *E_i_* be the excitation level of photoreceptors of type *i*. A function *M*(*E*_1_, *E*_2_, *E*_3_) is a color‐opponent mechanism if it is a linear combination of the photoreceptor excitation values.

(5) $$M\left(E_{1},E_{2},E_{3}\right)=c_{1}\cdot E_{1}+c_{2}\cdot E_{2}+c_{3}\cdot E_{3},$$

such that

(6) $$c_{1}+c_{2}+c_{3}=\;0.$$

Any combination satisfying Equations (5) and (6) is a color‐opponent mechanism (Chittka, 1992).

**Figure 2:**
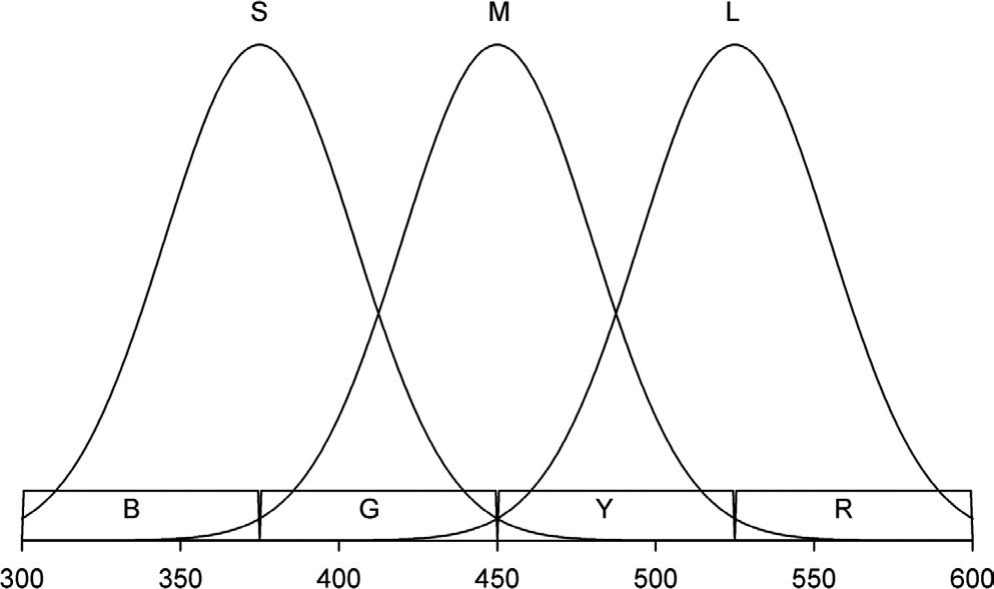
Photoreceptor S captures roughly light on segments B and G, M captures light on G and Y and L on Y and R. As a result, the difference between the number of photons captured by photoreceptors L and M can be approximated by (Y + R) − (G + Y) = R − G. Likewise, the difference between the amount of light captured by photoreceptors M and S can be approximated by (G + Y) − (B + G) = Y − B (see text for details)

Consider a trichromatic species. It has three photoreceptor types, *S*, *M* and *L*, with maximum sensitivity to light of wavelength *λ_S_*, *λ_M_* and *λ_L_*, respectively. Assume that *λ_S_*, *λ_M_* and *λ_L_* are evenly distributed, as in Figure 2. If this is the case, the rate at which photoreceptors of type *i* (*i* = *S*, *M* and *L*) absorb photons, and hence their excitation value, *E_i_*, can be approximated by:

(7a) $$E_{S}\approx B+G$$

(7b) $$E_{M}\approx G+Y$$

(7c) $$E_{L}\approx Y+R$$

Obviously, this is a very rough approximation. It assumes rectangular, rather than bell‐shaped, photoreceptor sensitivity curves, and a linear relationship between quantum catches (photon absorption) and photoreceptor excitations. Nevertheless, with these approximations,

(8a) $$E_{L}‐E_{M}\approx R‐G$$

(8b) $$E_{M}‐E_{S}\approx Y‐B$$

Comparing Equations ((4a), (4b), (4c)) and ((8a), (8b)), we see that the coordinates *Z*_1_ and *Z*_2_ approximate two color‐opponent mechanisms. The idea behind Endler's (1990) segment classification scheme, therefore, was to construct two species‐independent quantities that approximate the difference in excitation values between three consecutive photoreceptor types (*S* vs. *M* and *M* vs. *L*). To generalize the approach, it suffices to change the number of segments into which we divide the range (*λ*_1_, *λ*_2_) and the number of coordinates. Specifically, if a species has *n* photoreceptor types, we divide the range (*λ*_1_, *λ*_2_) in *n* + 1 equally sized segments. The *i*‐th segment corresponds to the wavelength range (*λ*_i−1_, *λ*_i_), with *λ*_i_ = *λ*_1_ + *i*·(*λ*_2_ − *λ*_1_)/(*n* + 1) and has relative brightness *S*_i_ = *β*(*λ*_i−1_, *λ*_i_)/*β*(*λ*_1_, *λ*_2_). The coordinates corresponding to a reflectance spectrum in the NSC model are the set of *n *− 1 chromatic coordinates,

(9a) $$Z_{i}=S_{i+2}‐S_{i},\;\text{with}\;i=1,2\ldots \;n‐1$$

and an achromatic (brightness) coordinate

(9b) $$Z_{n}=b\left(l_{1},l_{2}\right)\;/b_{m}\left(l_{1},l_{2}\right).$$

For the particular case of a trichromatic species, with *n* = 3, Equations (9a) and (9b) revert to Equation (4). We provide the NSC calculator, implemented in an Excel file as an example (Appendix S1). Users must adapt it to their needs, modifying (if necessary) the values of *λ*_1_ and *λ*_2_, the spacing between reflectance measurements and the spectral distribution of incident light.

### 3.2 Model extensions

We present two possible modifications of the model (recommended by John Endler). For simplicity, we present the modifications for the trichromatic version, but their generalization is straightforward. The first one consists in normalizing the chromatic components of the NSC model:

(10a) $$Z_{1}=2\cdot \left(R‐G\right)/\left(R+G\right)$$

(10b) $$Z_{2}=2\cdot \left(Y‐B\right)/\left(Y+B\right)$$

(10c) $$Z_{3}=\beta \left(\lambda_{1},\lambda_{2}\right)/\beta_{m}\left(\lambda_{1},\lambda_{2}\right).$$

The rationale for this normalization is that, if *Z*_1_ and *Z*_2_ are not scaled, model predictions can have artifacts depending upon the relative brightness of *R* + *G* relative to *Y* + *B*. While this statement is undoubtedly correct, this normalization introduces its own problems when *R* + *G* or *Y* + *B* are very small – which can magnify irrelevant differences. (Note that the denominator in the definition of *Z*_1_ cannot be close to 0, unless there is no light). For this reason, we provisionally adhere to the version of the NSC model specified by Equations ((4a), (4b), (4c)) and ((9a), (9b)).

The second modification has to do with the introduction of the brightness component. As presented above, the perceptual distance between two colors predicted by the NSC model is simply the Euclidean distance between their loci – coordinates (*Z*_1_, *Z*_2_, *Z*_3_). Some species, however, may not use brightness information, or do it only rarely (see discussion). For this reason, it might be better to give less weight to the brightness component than to the chromatic components. This can easily be done with the help of a free parameter, *α*. Let (*Z*_11_, *Z*_12_, *Z*_13_) and (*Z*_21_, *Z*_22_, *Z*_23_) be the loci of two colors. The perceptual distance between the two colors could be computed as:

(11) $$d_{12}=\sqrt{\left[{\left(Z_{11}‐Z_{21}\right)}^{2}+{\left(Z_{12}‐Z_{22}\right)}^{2}+\alpha \cdot {\left(Z_{13}‐Z_{23}\right)}^{2}\right]}$$

In this formulation, Endler's (1990) segment classification scheme corresponds to *α *= 0, and our proposed modification to *α *= 1.

### 3.3 Comparing the NSC to color discrimination models

According to the color‐opponent coding model (Backhaus, 1991), each color is associated to a point on a plane (its locus) and the perceptual distance between two colors is the distance between their loci, calculated according to the city‐block metric. In this model, the locus of a color is the point (*A*, *B*), with.

(12a) $$A=\;‐9.86\cdot E_{S}+7.70\cdot E_{M}+2.16\cdot E_{L}$$

(12b) $$B=\;‐5.17\cdot E_{S}+20.25\cdot E_{M}‐15.08\cdot E_{L},$$

The coefficients of *E*_L_ in Equation (12a) and *E*_S_ in Equation (12b) are much smaller (in absolute value) than the other coefficients in their respective equations, and therefore with the help of Equations ((4a), (4b), (4c)) and ((8a), (8b)) we see that

(13a) $$A\approx Z_{2}$$

(13b) $$B\approx \;‐Z_{1}$$

The color hexagon model is, in many respects, similar to the color‐opponent coding model. It differs from it in that it uses Euclidean distances rather than the city‐block metric to estimate perceptual differences and in the choice of color‐opponent mechanisms. Specifically, the locus of a color in the color hexagon model is the pair of color‐opponent mechanisms (Chittka, 1992):

(14a) $$X=\sqrt{3}\cdot \left(E_{L}‐E_{S}\right)/2$$

(14b) $$Y=E_{M}‐\;0.5\cdot \left(E_{S}+E_{L}\right),$$

These mechanisms are, in a sense, optimal: they maximize (subject to certain constraints) the spread and hence differentiability of color loci (Chittka, 1992). They are unrelated to the coordinates *Z*_i_ of the NSC model.

The original formulation of the receptor noise‐limited model calculates perceptual distances without assuming that colors are represented by a point in some space (Vorobyev & Osorio, 1998). But the distances predicted by the model correspond to the Euclidean distances between color loci in some space. For trichromatic species, the coordinates of the color loci are (Hempel de Ibarra et al., 2001).

(15a) $$X=A\cdot \left(E_{L}‐E_{M}\right)$$

(15b) $$Y=B\cdot \left(E_{S}‐\;\left(a\cdot E_{L}+b\cdot E_{M}\right)\right),$$

where *A*, *B*, *a* and *b* depend on the species under study (Hempel de Ibarra et al., 2001). The coordinate *X* in Equation (15a) is therefore similar to *Z*_1_ in the NSC model, but *Y* is unrelated to the NSC model coordinates.

To summarize, color discrimination models can be interpreted as assigning a locus in a space to each color and estimating perceptual differences between colors as the geometric distance between their corresponding loci. Model predictions differ in how they allocate loci to spectral distribution functions and the metric they use to compute distances, but in all cases the coordinates of the color loci correspond to color‐opponent mechanisms. The NSC model shares the logic of color discrimination models, except that it adds a brightness component to the set of color‐opponent mechanisms. And, of course, coordinates *Z*_1_ to *Z*_n−1_ only loosely approximate color‐opponent mechanisms – since photoreceptor sensitivities are not involved in their calculation.

### 3.4 Goodness of fit of NSC predictions to behavioral data

We now compare the predictions of the NSC model with those of traditional color discrimination models (color‐opponent coding model, color hexagon and receptor noise‐limited model) for two published data sets. In both cases, we take the predictions of alternative models and the behavioral data from the original publication, and simply calculate color distances according to the NSC model to check which color distance measure best correlates with performance in the experiment. In the NSC calculations, we use the standard D65 illuminant (Wyszecki & Stiles, 1982) for *D*(*λ*) in Equation (2) and the trichromatic version of the model.

In the first study bumblebees, *Bombus terrestris*, foraged in an experimental arena with two types of artificial flowers. Some flowers contained a nectar reward, others quinine. The color of the flowers was associated with their reward. All color pairs had similar perceptual distances according to the color hexagon model (0.05 hexagon units), but clearly different distances according to the color‐opponent coding model and the receptor noise‐limited model, in both linear and logarithmic versions. The probability that bees landed on nectar flowers increased with the distance predicted by the color‐opponent coding model and receptor noise‐limited model, but the correlation was far from perfect. There was evidence that bees based their foraging choices on both chromatic and achromatic information (Telles & Rodríguez‐Gironés, 2015).

**Figure 3:**
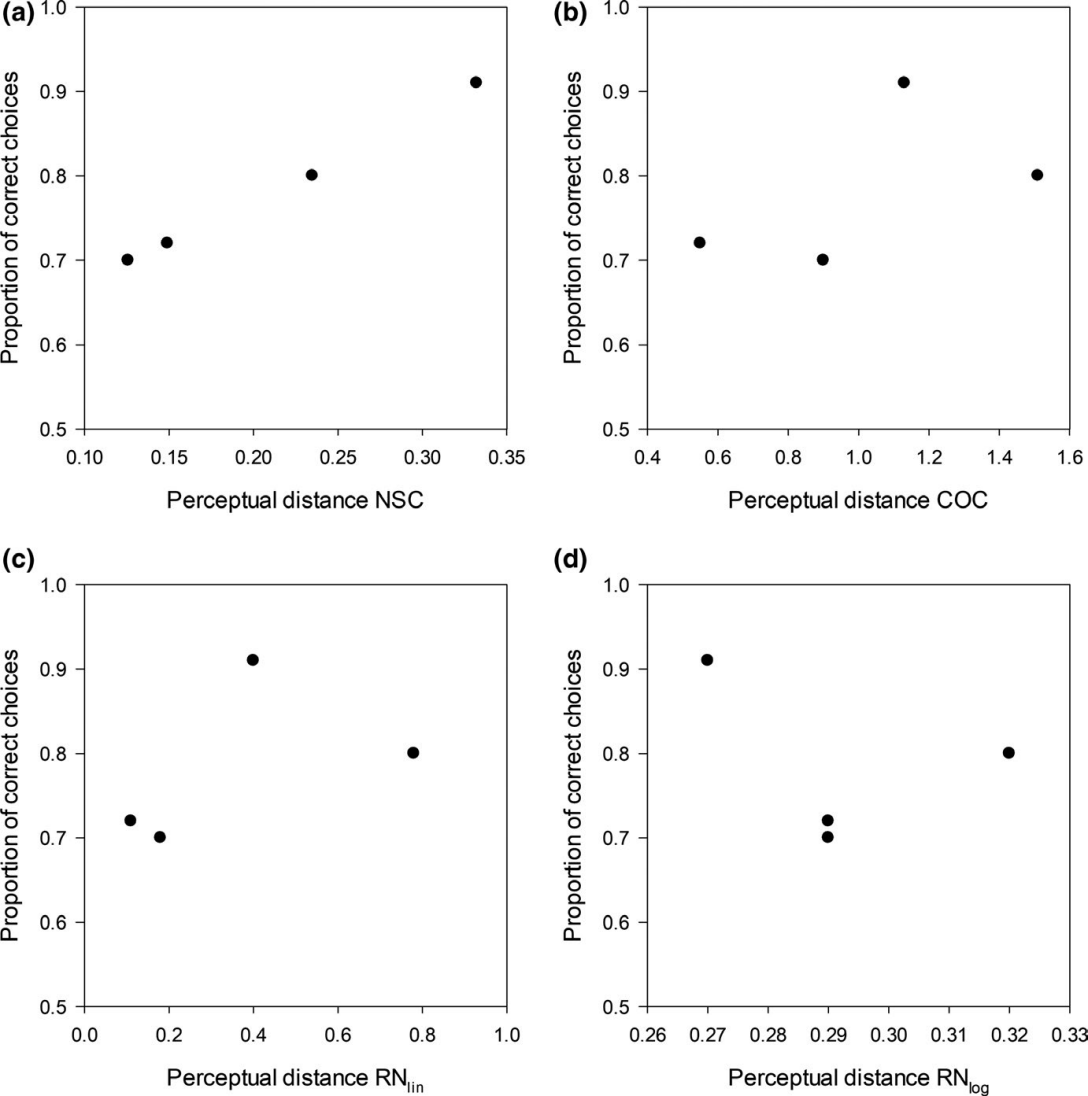
Bumblebees were trained to discriminate between four color pairs. The figure shows the relationship between performance and color distances predicted by different models: NSC (a), COC (b), linear and (c) logarithmic (d) versions of the RN model – colors were chosen so that the CH distance was the same for all pairs (0.05 hexagon units)

Figure 3 shows the relationship between bee performance and color distances, as predicted by the different models – including the NSC model, which provides the best fit to the data. The rank correlation (Spearman's R) between the proportion of correct choices and perceptual distances was 0.60 for the color‐opponent coding model, 0.65 for the hexagon model, −0.25 for the logarithmic version of the receptor noise‐limited model, 0.60 for the linear version, and 1 for the NSC (Figure 3). The importance of including the brightness component in the calculations of color distance is highlighted by the fact that, if we use the original segment classification scheme – which, for the trichromatic case, is equivalent to the NSC model without brightness component, *α *= 0 in Equation (11) – the rank correlation drops to −0.2. Finally, the version of the NSC model with normalized chromatic components (Equation (10a), (10b), (10c)) leads to a rank correlation of 0.80 – intermediate between those of the color hexagon and the standard NSC model.

**Figure 4:**
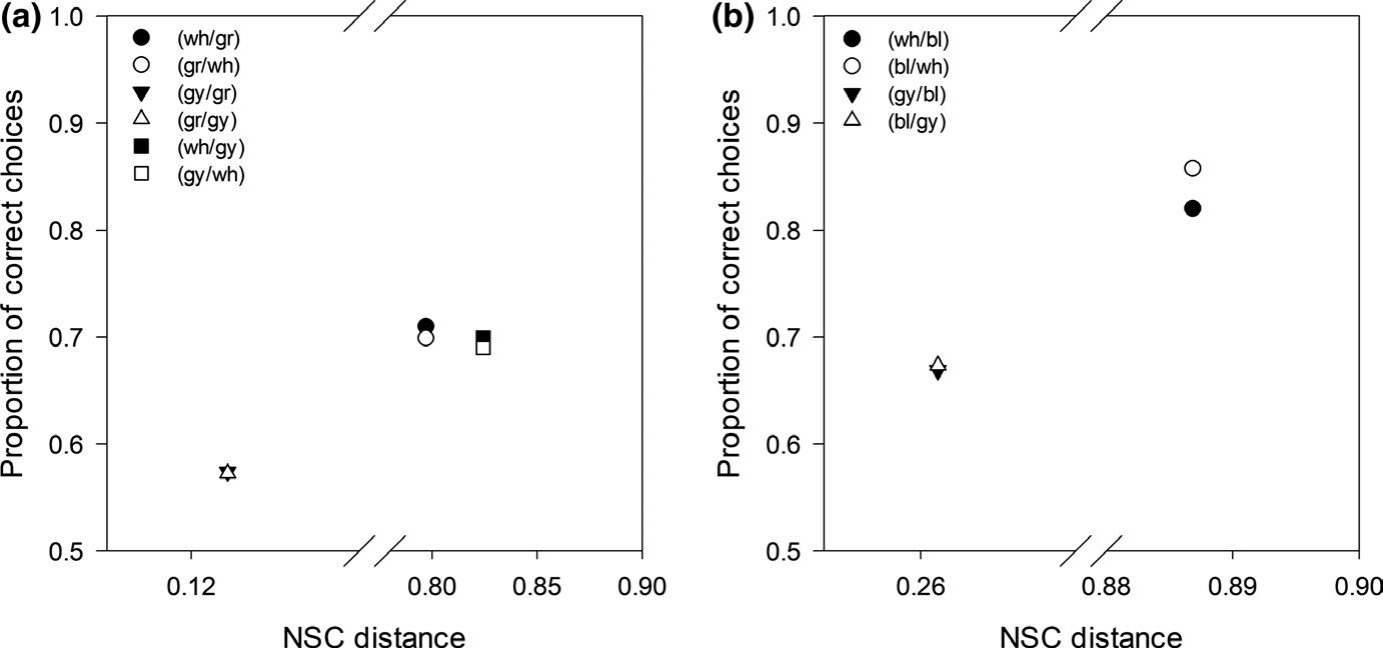
Experimental results of honeybees for different target/background color combinations tested in Hempel de Ibarra et al. (2000) and their relationship with the normalized segment classification distances (NSC). Proportion of correct choices for the detection of the stimuli in reciprocal target/background combinations of (a) ultraviolet‐ reflecting white (wh), gray (gy) and green (gr) and (b) ultraviolet‐reflecting white, gray and blue (bl)

In a second study, honeybees *Apis mellifera* foraging for nectar entered a Y maze. In one of the arms, a colored circle against the background indicated the presence of nectar. In the other arm, where there was no colored circle, there was no nectar either. The authors tested the ability of bees to detect the presence of the colored circle using five color pairs. Within each pair, one color served sometimes as background and sometimes as target. Because, for each pair, color distances and bee performance were independent of which of the two colors served as target and which as background (Hempel de Ibarra et al., 2000), we computed the average performance of bees across the two conditions. None of the models tested managed to predict the results of the experiment (Hempel de Ibarra et al., 2000). The rank correlation (Spearman's R) between the proportion of correct choices and perceptual distances ranged from −0.48 to 0.48 for all the models tested. For the trichromatic version of the NSC, however, the rank correlation was 0.90 (Figure 4a,b). As in the previous case, removing the brightness component leads to a drastic deterioration of the goodness of fit. For the original segment classification scheme, *α *= 0 in Equation (11), the rank correlation was only 0.20. Using Equation ((10a), (10b), (10c)) instead of Equation ((4a), (4b), (4c)) (normalized chromatic components) the rank correlation was 0.60 – once again better than for traditional models, but not as high as when we used Equation ((4a), (4b), (4c)).

## 4 DISCUSSION

The NSC model allows us to estimate color differences irrespective of the viewer species. It has strengths and limitations. Among the latter, it is unable to account for any degree of color constancy (Chittka et al., 2014; Olsson et al., 2016), and it can assign positive color distances to metameric pairs – pairs of colors that have different spectral power function but produce the same excitation values in the photoreceptors and that, therefore, cannot possibly be distinguished (Cohen & Kappauf, 1982).

### 4.1 Model use

Color is not a physical property of objects; it is a perception. Without a viewer, there is no color (Kelber et al., 2003). Because the NSC model ignores the visual system of the observer, it can be argued that it is not a color discrimination model, but a method to compare the spectral distribution of light beams (Grill & Rush, 2000; Smith, 2014).

Kemp et al. (2015) suggest that the biological question should determine the type of model we use. In particular, they believe that we should use species‐independent models, such as the NSC, mainly to study “situations that are largely independent of sensory systems” (Kemp et al., 2015; p. 711), or when the viewers of the trait under study are very diverse or unknown (or both). In theory, these models give us information about how different two (or more) spectral curves are, but they tell us nothing about perceptual differences, since the visual system of viewers is excluded from their formulation. As a result, when using these models, “it is important to acknowledge the limitations to inferences about color ecology and evolution” (Kemp et al., 2015; p. 714). When we are interested in how different certain colors appear to viewers, we are told, we should use models that include sensory information about the viewer.

While we endorse this view in general terms, it seems to us that things are not always clear‐cut and that the rule should not be rigidly enforced. For instance, we may be interested in how different from each other the colors in a set look to a diverse array of viewers. It may sometimes be possible to use different models for the different species involved, but it may be difficult (if not impossible) to compare the results between species: a distance of 0.1 can be “large” for one model (color hexagon) and “small” for others (color‐opponent coding model, receptor noise‐limited model). If a single species‐independent model can estimate how similar colors appear to the different viewers, it may be preferable to use such model.

As ecologists, our main question will often be how difficult it is, for animals, to discriminate between colors, or if animal assemblages have similar preferences (e.g., Buide et al., 2015; Reverté et al., 2016). The claim that we should use species‐specific models, including sensory information about the viewer, to estimate color differences is based on the implicit assumption that the perceptual distances predicted by species‐specific models correlate better with the ability of animals to discriminate colors than the predictions of species‐independent models. However, as we have seen above, for at least two published datasets the correlation between the proportion of correct choices and the perceptual distances predicted by the NSC model was higher than for alternative, species‐specific, models.

It is clearly too early to know whether and when the predictions of the NSC model describe the discrimination ability of animals better than the predictions of other models: the two datasets we have explored might just be an exception. It is only through systematic studies, comparing the ability of species to discriminate between many color pairs with the predictions of available models, that we will be able to learn which model is more appropriate for which species, experimental setup, or color range. While we wait for these experiments to be done, when making inferences about color ecology and evolution it will be good practice to remember that every visual model has limitations: regardless of whether it includes, or not, information about the sensory system of the viewer.

### 4.2 Chromatic and achromatic information

Another reason why vision scientists may not consider the NSC a color discrimination model is that, according to this model, brightness differences affect color distances. For vision scientists, color and brightness are orthogonal concepts: the color of an object is, by definition, independent of the amount of light it reflects or emits (Kelber et al., 2003; Kemp et al., 2015; Menzel, 1979). It is for this reason that the output of color discrimination models (color‐opponent coding, color hexagon, receptor noise‐limited models) is, as much as possible, independent of brightness differences.

The decision to factor out achromatic information from color discrimination models is not just a whim. Chromatic information – information about whether a photoreceptor type is more or less excited than another – is processed through different channels than achromatic information – information about the average excitation level of photoreceptors – both in vertebrates and in invertebrates (Livingstone & Hubel, 1988; Nassi & Callaway, 2009; Paulk et al., 2008).

The fact that different channels process chromatic and achromatic information, however, does not imply that they are perceptually independent. Primate brains, for instance, combine chromatic and achromatic cues at some stage before the final assessment of visual information (Abramov & Gordon, 1994; Burns & Shepp, 1988; Nagy, 1999). Thus, although we can judge the brightness of a colored stimulus, or the amount of blue/yellow and green/red in the color, we do not perceive brightness, blueness and greenness independently (the way we perceive the color and scent of a flower), but as a single percept.

In our view, there is not enough information to determine whether nonhuman animals perceive brightness as independent from color – and we see no obvious reason to assume that this is the case. When brightness differences are redundant, because the chromatic information is sufficient for stimulus discrimination, animals may ignore the brightness components. For instance, while a recent study failed to train honeybees, *Apis mellifera*, to associate achromatic stimuli with a reward, despite the large achromatic differences between stimuli and background (Ng et al., 2018), a previous one using a very similar experimental design (Hempel de Ibarra et al., 2000), managed to train honeybees to associate an achromatic stimulus with nectar (color hexagon distance 0.02, 70% correct choices). When testing color preferences of trained flies (*Eristalis tenax*) with yellow spots differing in brightness and UV‐reflection properties, Neimann et al. (2018) showed that the color brightness had an impact on attractiveness of yellow spots. The dark yellow colors triggered the extension of the proboscis significantly more often than bright yellow colors. The authors also trained flies with green artificial flowers varying in brightness. During dual choice tests, in those cases where the differences in brightness between the two colors were strong, bright colors were more attractive for landing than dark ones, irrespective of the trained color (Neimann et al., 2018). Indeed, the reason why the NSC model fits better behavioral data (from Hempel de Ibarra et al., 2000; Telles & Rodríguez‐Gironés, 2015) than alternative models is precisely because the NSC model includes achromatic differences.

## CONFLICT OF INTEREST

Authors disclose any potential sources of conflict of interest.

## AUTHOR CONTRIBUTIONS

**Miguel Angel Rodríguez‐Gironés:** Conceptualization (lead); data curation (lead); formal analysis (lead); funding acquisition (lead); investigation (lead); methodology (lead); project administration (lead); validation (equal); visualization (equal); writing–original draft (equal); writing–review and editing (equal). **Francismeire Jane Telles:** Data curation (supporting); formal analysis (supporting); investigation (supporting); methodology (supporting); validation (equal); visualization (equal); writing–original draft (equal); writing–review and editing (equal).

## Supporting information

Supplementary Material

## Data Availability

Data used in this manuscript can be found in previous published papers or open databases, as stated in the main text and figure legends.

## APPENDIX 1

### NSC CALCULATOR

We provide Appendix S1 an Excel file that can be used to calculate NSC distances between pairs of stimuli. The file contains three sheets, corresponding to the dichromatic, trichromatic and tetrachromatic versions of the model. We explain here the workings of the trichromatic version so that users can modify it to suit their needs. The logic of the di‐ and tetrachromatic versions is essentially identical.

Column A, labelled “wl” contains the wavelength of light corresponding to each row. In the file we provide, wavelength ranges between 300 and 700 nm, and consecutive rows differ by 5 nm intervals. Users interested in different wavelength ranges or collecting their reflectance data with more or less detail will have to adapt the NSC calculation to their data structure.

Column B, labelled D65, must contain the relative amount of light – in photon fluxes, not in energy per unit time – available at each wavelength. For the NSC, we have included the D65 standard, which works well for daytime applications in open spaces. Users working with crepuscular or nocturnal species, living in forests or underwater, will need to measure light availability in the environment of interest and include the relevant data in this column.

Columns C and D, labelled C1 and C2, must contain the spectral reflectance data for the colors of interest. The values in the NSC correspond to hypothetical colors, with no particular meaning or relevance.

Columns E and F, labelled PhF_C1 and PhF_C2, contain the product of light availability (column B) times spectral reflectance (column C or D) for each wavelength. These columns therefore contain (ignoring scatter due to turbid water, fog, smoke and the like) the spectral distribution of light impinging the eye when viewing an object of color C1 (column E) or C2 (column F) – Equation (2).

From these data we compute, for each color, *β*(*λ*_1_, *λ*_2_) and *β*_m_(*λ*_1_, *λ*_2_). They are labelled as “beta” and “betaMax” in the NSC calculator, and the values for C1 and C2 are in cells I2 and J2 (for *β* (*λ*_1_, *λ*_2_)) and I3 and J3 (for *β*_m_(*λ*_1_, *λ*_2_)). Remember that *β* (*λ*_1_, *λ*_2_) is the total amount of light reflected by the object, and *β*_m_(*λ*_1_, *λ*_2_) the maximum amount of light an object can reflect (the light present in the environment). We now divide the spectrum in four equally sized intervals. In our case, these intervals are 300–400 nm, 405–500 nm, 505–600 nm and 605–700 nm. As a visual aid, we have given a different color to each interval. We now compute, in cells I4 to I7 for C1 and J4 to J7 for C2, the proportion of the light reflected that corresponds to each of these four intervals: S1, S2, S3 and S4 (corresponding to *S*_1_, *S*_2_, *S*_3_ and *S*_4_ in the model formulation, Equation (9a), (9b)). In the sheet for dichromatic species, the wavelength range is divided in three intervals and only three quantities, S1, S2 and S3, are computed. For tetrachromatic species, on the other hand, we divide the wavelength range in five intervals and calculate S1, S2, S3, S4 and S5.

We now proceed to calculate, for C1 and C2, the coordinates determining the locus of each color in the NSC space: *Z*_1_, *Z*_2_, and *Z*_3_ (equation (9a), (9b)). The distance between C1 and C2 according to the NSC model (cell L14) is simply the Euclidean distance between their loci.
